# Toward Model Building for Visual Aesthetic Perception

**DOI:** 10.1155/2017/1292801

**Published:** 2017-11-15

**Authors:** Jianli Liu, Edwin Lughofer, Xianyi Zeng

**Affiliations:** ^1^College of Textiles and Clothing, Jiangnan University, Wuxi 214122, China; ^2^Department of Knowledge-Based Mathematical Systems, Johannes Kepler University Linz, 4040 Linz, Austria; ^3^Université Lille Nord de France, 59000 Lille, France; ^4^ENSAIT, GEMTEX, 59056 Roubaix, France

## Abstract

Several models of visual aesthetic perception have been proposed in recent years. Such models have drawn on investigations into the neural underpinnings of visual aesthetics, utilizing neurophysiological techniques and brain imaging techniques including functional magnetic resonance imaging, magnetoencephalography, and electroencephalography. The neural mechanisms underlying the aesthetic perception of the visual arts have been explained from the perspectives of neuropsychology, brain and cognitive science, informatics, and statistics. Although corresponding models have been constructed, the majority of these models contain elements that are difficult to be simulated or quantified using simple mathematical functions. In this review, we discuss the hypotheses, conceptions, and structures of six typical models for human aesthetic appreciation in the visual domain: the neuropsychological, information processing, mirror, quartet, and two hierarchical feed-forward layered models. Additionally, the neural foundation of aesthetic perception, appreciation, or judgement for each model is summarized. The development of a unified framework for the neurobiological mechanisms underlying the aesthetic perception of visual art and the validation of this framework via mathematical simulation is an interesting challenge in neuroaesthetics research. This review aims to provide information regarding the most promising proposals for bridging the gap between visual information processing and brain activity involved in aesthetic appreciation.

## 1. Introduction

The ability to appreciate the aesthetic qualities of natural and manmade items is one of the universal characteristics of humanity. Neuroaesthetics is the discipline of investigating how beauty activates aesthetic perception and appreciation. It is situated at the intersection of psychological aesthetics, neuroscience, and human evolution [1]. The main objective of neuroaesthetics is to “characterize the neurobiological foundations and evolutionary history of the cognitive and affective processes involved in aesthetic experiences and artistic and other creative activities” [2]. It has taken nearly two decades for neuroaesthetics to establish itself as a serious discipline concerned with the scientific investigation of aesthetics from a neurobiological perspective [3]. Most previous neuroaesthetic studies have focused on the investigation of the neural mechanisms underlying aesthetic appreciation as well as those factors that make certain stimuli—such as the visual arts, dance, the human face, and music—beautiful or attractive. Many studies have explored the neural foundations of aesthetic perception and appreciation, leading to the development of models for aesthetic appreciation and judgement. This review primarily aims to summarize the current models for aesthetic appreciation and judgement, to analyze the strategies used to develop these models, and to compare the components included in each model.

The neural correlates of visual aesthetic perception have been comprehensively investigated using functional magnetic resonance imaging (fMRI), electroencephalographic (EEG) recording, and transcranial magnetic stimulation (TMS). Subsequently, models have been constructed to promote interdisciplinary understanding and communication among different disciplines involved in the study of the mind [4].

Researchers in the fields of neuroscience, cognitive science, and information science are faced with two major challenges concerning neuroaesthetics. (1) Are there putative neural networks in our brain that are responsible for visual aesthetic appreciation and aesthetic judgement? (2) Where in the brain does neural activation associated with aesthetic responses to visual stimuli occur? Such questions must be answered in order to develop more precise mathematical models of visual aesthetic appreciation. The general framework for the neural underpinnings of visual aesthetics guided by visual neuroscience reveals the prospects for cognitive neuroscience in this field. This can be considered the current working model of how the neuroscience of visual aesthetics might be mapped [11].

## 2. The Neural Foundation of Visual Aesthetic Appreciation

Significant research effort has been devoted to investigating the neural underpinnings of the aesthetic experience of visual art using functional neuroimaging. The primary aim of such research is to reveal which brain areas, neural circuits, and mechanisms are involved in aesthetic responses to visual art. Areas most commonly activated in aesthetic appreciation tasks include the orbitofrontal cortex (OFC), anterior insula (frontal insula, FI), and anterior cingulate cortex (ACC) [15]. According to the theory of measurable brain activity proposed by Nadal and Pearce (2011), the reward circuit is involved in the aesthetic appreciation of painting, music, and dance. The reward circuit includes the cortical (anterior cingulate, orbitofrontal, and ventromedial prefrontal) and subcortical (ventral tegmental area and nucleus accumbens) regions and circuit regulators (amygdala, thalamus, and hippocampus), with the exception of the visual cortex [16].

Vessel and colleagues discovered that regions in the medial prefrontal cortex, which is part of the default mode network (DMN), were positively activated during the aesthetic appreciation of visual art [17]. Further research indicated that DMN regions exhibited activation during visual aesthetic response experiments [18]. In neuroscience, the DMN is most commonly active when a person is not focusing on the outside world and the brain is at wakeful rest [19, 20]. However, Cela-Conde and colleagues noted that aesthetic appreciation relies on the activation of two different networks, an initial and a delayed aesthetic network—a conclusion supported by neuroimaging analyses of functional connectivity during visual aesthetic experiences [21, 22]. Cela-Conde and colleagues also suggested that the DMN may correspond primarily to the delayed aesthetic network.

One of the primary goals of neuroaesthetics is to uncover the neural foundations of aesthetic perception [23]. Less than two decades after the inception of neuroaesthetics, the burgeoning field of visual aesthetics perception continues to grow in interest and momentum [4]. Some relevant research to date has indicated that a distributed set of brain regions involved in perceptual, cognitive, and emotional processing is activated when an aesthetic experience occurs. A recent meta-analysis by Skov and colleagues revealed that the occipital lobes, anterior insula, and posterior cingulate cortex are activated during the viewing of paintings [24]. The activated brain regions are also involved in processing early and intermediate visual information, such as the perception of objects (fusiform gyrus), scenes (parahippocampal gyrus), and the experience of emotion (the anterior insula) [25].

Two research groups showed that the content of the visual artwork affects aesthetic judgement and appreciation via fMRI experiments [5, 26]. Such experiments revealed that the fusiform face area was activated to a greater degree when participants viewed painted portraits, while the parahippocampal place area was activated to a greater degree when participants viewed natural scenes. A recent meta-analysis of 47 fMRI experiments conducted by Boccia et al. revealed that aesthetics-related neural systems and regions are widely distributed throughout the brain [5]. The 27 regions showing consistent activation in the research of Boccia et al. are listed in Table 1. BA and Hem are the abbreviation for Brodmann areas and hemisphere, respectively.

As listed in Table 1, there are 27 brain regions involved in aesthetics appreciation of visual art. To some extent, the brain regions as mentioned in Table 1, powerfully supporting that aesthetic perception, appreciation, and judgement have neural foundations. In other words, these findings are credible answers to the two major challenges concerning neuroaesthetics as mentioned in Section 1. Numerous additional studies have implicated these regions in aesthetic appreciation and judgement as well (Bohrn et al., 2013 [27, 28]; Jacobsen et al., 2006; Kawabata and Zeki, 2004 [25, 29]; Munar et al., 2012b [24]; Zeki et al., 2014). However, some outstanding questions remain regarding the biological bases of aesthetic appreciation, such as those concerning the roles of the DMN and different regions of the extended reward circuitry [30].

## 3. Models of Aesthetic Appreciation

Over the past decade, several neutrally based and neutrally inspired models of aesthetic perception have been proposed. Although the internal structures of the models differ, the aims are very similar.

### 3.1. The Neuropsychological Model

Chatterjee proposed a general framework for the neural underpinnings of visual aesthetics guided by visual neuroscience [6, 30]. In this framework, visual information processing associated with early feature extraction and object recognition is completed in the early visual layer and intermediate visual layer, respectively. Early features such as color, orientation, and shape are processed in the occipital cortex, following which they are integrated in the intermediate vision layer to form a larger visual target. Visual attention and representation functions are engaged in and assigned into two parallel layers. The final stages of visual processing involve the generation of emotional responses and decision-making. The outputs of the emotional response layer include choosing between matched pairs such as liking versus wanting.

**Figure 1:**
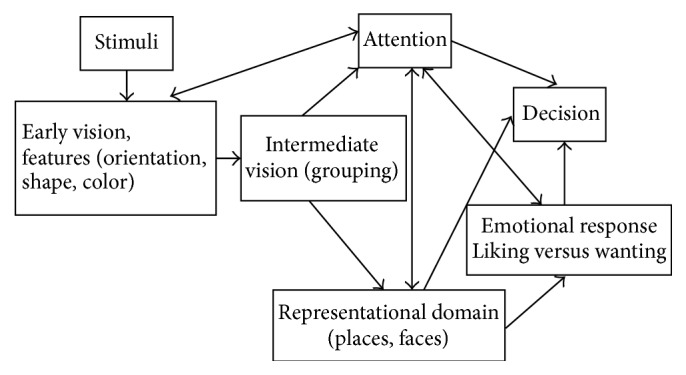
Chatterjee's model of the neural underpinnings of visual aesthetics, reproduced from Chatterjee, 2004.

The working model of Chatterjee's proposal is shown in Figure 1, which emphasizes the functions of the visual system. For early vision, Chatterjee argued that occipital cortex and frontal-parietal attentional circuits were the most important neural foundation [6]. The simple components, such as color, shape, location, luminance, and motion, will be processed by early and intermediate vision. The late vision that includes orbitofrontal cortex, insula, temporal pole, and ventromedial prefrontal cortex, is responsible for cognitive and emotional processing [31]. Cela-Conde et al. found that the main regions involved in Chatterjee model are occipital, frontal-parietal, temporal, frontal-parietal-temporal, medial temporal, medial and orbitofrontal, and subcortical region, which are the core regions of frontoparietal networks, ventral visual stream, feed-back/feed-forward links, anterior medial temporal lobe, medial and orbitofrontal cortices, and subcortical structures [32].

This model has been supported by the work of Basole et al. [33]. Visual attention modulates the spatial and temporal sensitivity of early perceptual filters and influences the selection of stimuli of interest, an essential step in visual perception [34]. Representation of some objects is also completed after the visual attention stage [35]. “Decision-making” is likely to refer to behavioral decisions. In Chatterjee's model, emotional components and some mnemonic domains related to previous personal history, such as faces and places, are also involved.

The neuropsychological model draws its main internal structures from vision cognition research. To some extent, Chatterjee's model can be explained by Nadal and Pearce's theory [36]. By combining the model in Figure 1 and the theory of measureable activity, we can deduce that (1) enhancement of low-level cortical sensory processing is the major function of the early and intermediate visual systems, (2) high-level top-down processing and activation of cortical areas involved in evaluative judgement are completed during the attention and decision-making stages, and (3) emotional responses and aesthetic appreciation engage the reward circuit.

The model proposed by Chatterjee is a “feed-forward” system involved in the processing of different attributes, providing a neurophysiological framework for visual aesthetics. However, if we aim to build a mathematical representation of the model proposed by Chatterjee, the functions of early/intermediate visual systems, visual attention, and representation must be translated into relevant aspects of computer vision, such as feature extraction and selection, target recognition, and scene classification.

### 3.2. The Information-Processing Model

**Figure 2:**
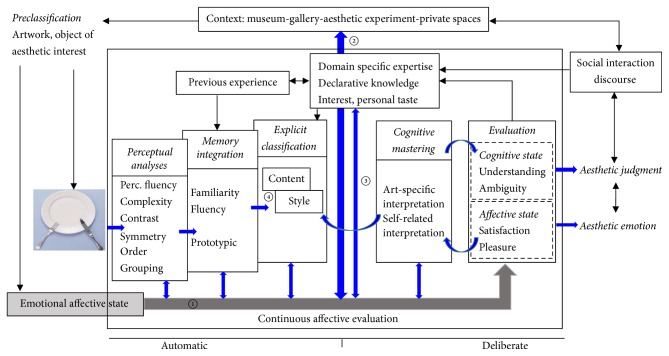
Model of aesthetic appreciation and aesthetic judgement, reproduced from Leder and Nadal, 2014.

Leder et al. proposed an influential framework of aesthetic experiences and aesthetic judgement in their cognitive model of information processing [7, 8]. This model was updated by Leder and Nadal in 2014 [3].  Figure 2 clearly demonstrates the flow of information through different circuits, a very similar process to the flow of signals in the brain. The internal structure of this model is supported by the research conclusion that aesthetic appreciation and judgement requires a distributed neural network of activation in the brain, rather than in a single neural region [5, 22, 24, 37]. The neural underpinnings for the information-processing model at least cover three functionally distinct sets of brain regions. The reward circuit is the first set, which is constituted by cortical (anterior cingulate, orbitofrontal, insular, and ventromedial prefrontal) and subcortical (caudate nucleus, substantia nigra, and nucleus accumbens) regions, as well as some of the regulators (amygdala, thalamus, and hippocampus) of the reward circuit [38, 39].

The model constructed by Leder and colleagues considers much the same elements as Chatterjee's model, though the crucial feature of context has been added [3]. This model is helpful for understanding the mechanisms underlying the involvement of cognitive and affective processes in the aesthetic appreciation of visual art. However, the translation of this complex framework into a mathematical model is very challenging because factors such as art content and style and the individual's previous experience and social interaction cannot be easily quantified.

With the development of empirical aesthetics over the last ten years, this model gained appeal with researchers in cognitive and information fields. Originally, the model aimed to provide an integrated description of the psychological processes involved in the aesthetic appreciation of art; therefore, it can be seen as an attempt to determine the psychological mechanisms and the contextual conditions that enable people's engagement with artworks to be valuable, affectively absorbing, and individually and socially meaningful experiences [40]. The model focused on, but not restricted to, modern art.

The model included a sequence of processing stages within the perceiver, flanked by constituting conditions. It was designed as an information-processing box model that summarized a variety of findings related to the way perception, knowledge, familiarity, expertise, style, and content, among others, influence the aesthetic experience of art. The model comprises five main processing stages: perception, implicit memory integration, explicit classification, cognitive mastering and evaluation, and continuous emotional evaluation.

### 3.3. The Mirror Model of Art

Tinio proposed the mirror model of art to describe the interface between making and viewing art [9]. According to the mirror model, the early stages of aesthetic processing correspond to the final stages of art making, while the late stages of aesthetic processing correspond to the initial stages of art making. The complete aesthetic experience of visual art, including both the creation and the appreciation of art, is the primary focus of this cognitive model. To some extent, the model is developed on the assumption that the two stages of creation and appreciation of art are related.

**Figure 3:**
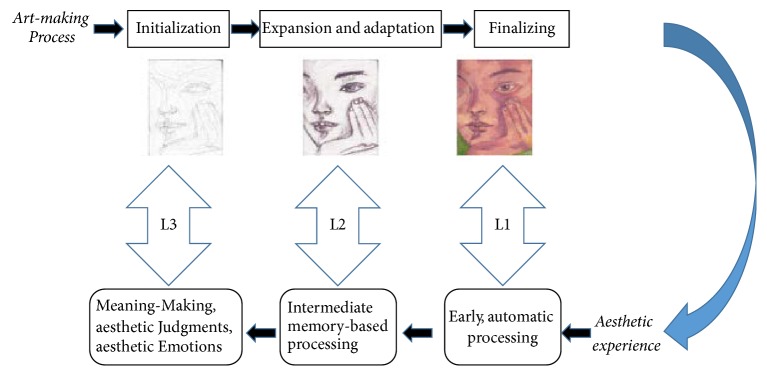
The mirror model of art, adapted from Tinio, 2013. Downloaded from the supplementary materials provided by Pablo P. L. Tinio from http://dx.doi.org/10.1037/a0030872.supp.

This model is very successful when artists appreciate their own works, as artists have a much deeper understanding of their work than anyone else because of their prior knowledge regarding its creation and the emotion expressed at the time [41].  Figure 3 illustrates the mirror model. The three processes represent the three levels of correspondence between art creation and art appreciation. The initial perception of many features of existing visual arts occurs automatically, while the creation of art will activate the artist's aesthetics knowledge and background experiences [9]. Mizokami et al. found that the left lingual gyrus and bilateral cuneus may be associated with aesthetic judgement of representational paintings through fMRI [29]. The researches of Vessel and his collaborators indicate that regions in the medial prefrontal cortex that are known to be part of the default mode network (DMN) were positively activated on the highest-rated trials in a task of art appreciation and creation [18]. Cattaneo et al. (TMS) found that the lateral occipital area, the left prefrontal cortex, and the right posterior parietal cortex play a fundamental role in the aesthetic appreciation of representational and abstract paintings through transcranial magnetic stimulation [42, 43].

Figure 3 converts the process of art creation into a formula. Mace and Ward adopted a different approach and noted that the physical, material, and procedural constraints associated with art creation mean that the creative art-making process has some common stages [44]. The artist initially constructs numerous sketches to demonstrate the idea that he or she considers appropriate for conveying particular emotions and/or information. The draft is further developed as details are highlighted during the expansion and adaptation stages. Finally, the artwork is refined and completed. Thus, the aesthetic experience of art is divided into three stages, similar to the theory proposed by Leder et al., which describes five stages.

### 3.4. The Quartet Model of Human Emotion

**Figure 4:**
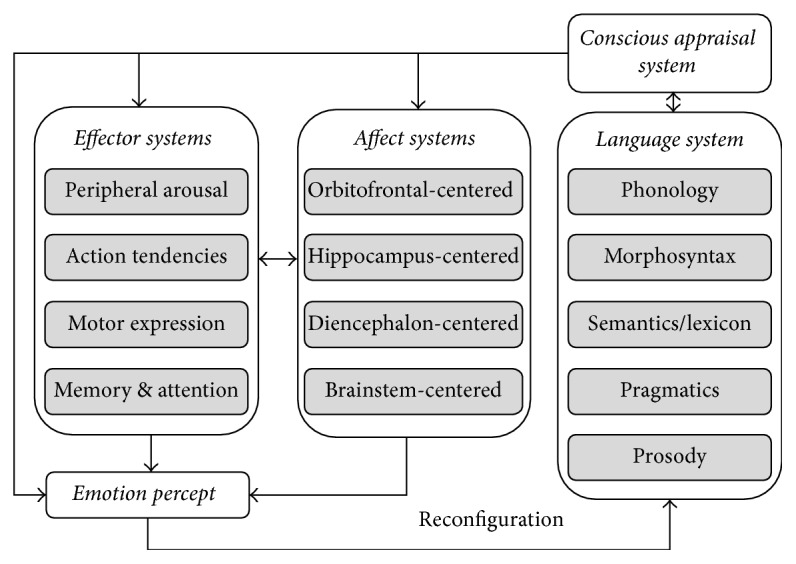
The emotion model proposed by Koelsch et al., reproduced from Koelsch, 2015.

Koelsch and colleagues proposed an integrative and neurofunctional framework for human emotions that includes four core emotional systems from the perspective of neural motion processing [10]: the effector, affect, language, and conscious appraisal systems. The quartet theory offers a comprehensive and anatomically detailed framework for understanding the neural correlates of human emotions. The model's structure is shown in Figure 4, which shows that emotion is not generated by isolated neural systems but by interactions among lower- and higher-level neural circuits. The four systems have a number of features in common; for example, they are all supported by complex neural circuits and interact with each other. Intriguingly, all four systems (brainstem-centered, diencephalon-centered, hippocampus-centered, and orbitofrontal-centered systems) comprise brain areas whose role in emotional processing is in addition to mediating other specific aspects of cognition [45]. The brainstem for emotional processes is ascending activation mediated mainly by numerous nuclei, which is the reticular formation and occupies the central portion of the brainstem [46]. The main components of the diencephalon are the dorsal and ventral thalamus, hypothalamus, epithalamus, habenular complex, pineal gland, and subthalamic nucleus [47]. The hippocampus consists of cortex and is a transitional mesocortex consisting of three to five layers [45]. The orbitofrontal cortex corresponds to Brodmann areas 47 and 11 and partly 10, which is related to the following affective aspects: automatic cognitive appraisal, generation of “somatic markers,” reward and punishment, and moral emotions [10]. These systems build upon each other, each one introducing new sophistication that allows a distinct class of emotional states to emerge [48].

The quartet theory is an auspicious starting point for shedding light on the multifaceted occurrence of affective experiences in relation to art and aesthetics [49]. The theory addresses the role of the four neuroanatomically distinct cerebral systems in emotion processing extensively, although it lacks proper integration with other fundamental sensory and perceptual systems and neural networks that also play a critical role in the emotional experience [50]. As such, the model is a valuable contribution to the field of affective neuroscience, perhaps pushing our understanding “one step closer to finding the elusive emotional brain” [51].

This model is also the first to consider the regulatory and communication functions of language as integral elements in emotion processing [52]. Although the role of language in emotional regulation warrants highlighting, such regulation is not based exclusively on linguistic processes [53]. Additionally, several modular psychological conceptions, such as learning and memory, antecedents of affective activity, emotion satiation, cognitive complexity, subjective emotions, and degree of conscious appraisal, are assigned to different affect systems in this theory. However, the hypothesis that modular psychological concepts such as affect and emotion can be assigned to a distinct subset of regional neural circuits remains questionable [54].

### 3.5. The Unifying Model of Visual Aesthetic Experience

**Figure 5:**
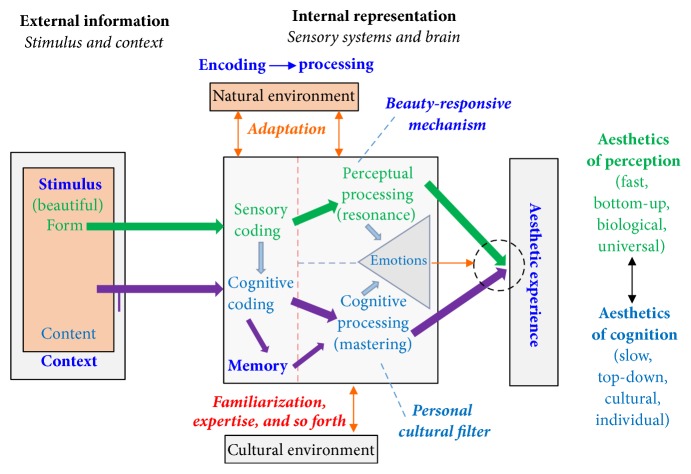
Model of aesthetic experience proposed by Redies, reproduced from Redies, 2015. Green arrows represent the perceptual channel, which is responsible for processing visual stimulus. Purple arrows represent the cognitive channel, which processes the content and context of the stimulus. The black dashed circle represents the joint action of the two channels.

Redies proposed a unifying model of visual aesthetic experience that combines the formalist and contextual aspects of aesthetics [11]. This dynamic model explains changes in the reception of artworks over time.  Figure 5 depicts Redies' model of visual aesthetic experience. The two circuits in the model represent aesthetic perception and cognition, which are processed in parallel by the perceptual and cognitive channels. The perceptual channel processes visual stimuli, while the cognitive channel processes the content and context of the stimulus. Resonance in the perceptual channel and mastering and positive evaluation in the cognitive channel are vital sections of emotion generation and aesthetic experience [55]. The model also considers the differences between the aesthetics of perception and cognition.

The neural mechanism responsive to beauty is activated in the visual brain regions, which predominantly proceeds the visual information in a bottom-up direction [56]. In the bottom-up mechanisms, the visual stimulus, such as a painted artwork, a natural scene, or an artificial pattern, will be captured by the fovea of the retina. And then, the retinal ganglion cells will project the encoded visual information through nerve fibers to the visual centers in the brain. Sensory coding and basic processing of visual information are mediated by neuronal mechanisms that are composed of the retina and visual brain. The medial and lateral subdivisions of the orbitofrontal cortex as well as subcortical stations are associated with affective motor planning (globus pallidus, putamen–claustrum, amygdala, and cerebellar vermis), whereas the motor, premotor, and supplementary motor areas, as well as the anterior insula and the dorsolateral prefrontal cortex, are the core of visual brain for aesthetic perception of visual stimulus [27]. After the comprehensive analysis of 93 neuroimaging studies of positive-valence aesthetic appraisal, Brown et al. pointed out that the visual brain engaged in aesthetic perception including the left inferior parietal lobule, fusiform gyri bilaterally, inferior frontal gyri bilaterally, hypothalamus, and caudate nuclei and amygdala bilaterally [15].

Stimulus and context are the two main components of external information in Figure 5. They are encoded and processed by the sensory and perceptual nervous systems, respectively. External information associated with an artwork is encoded by the nervous system and processed in the brain by the interaction of the perception and cognition channels. This interaction reflects the connectivity among neural networks in different brain areas related to aesthetic perception. The model was primarily developed for visual artworks, but its application to other senses has also been considered.

### 3.6. The Hierarchical “Feed-Forward” Model

**Figure 6:**
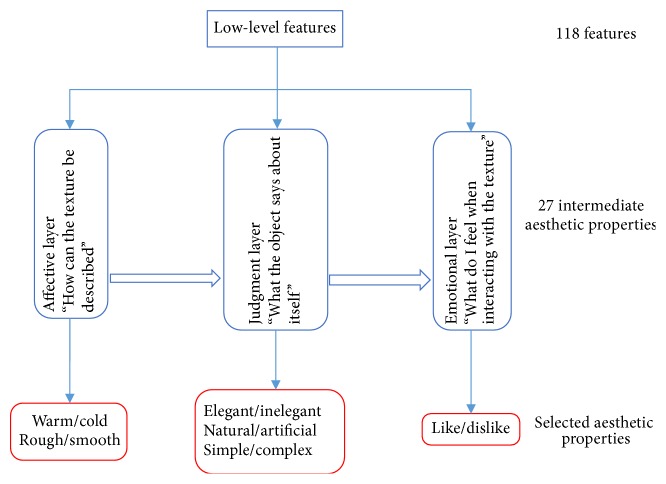
Hierarchical feed-forward model, reproduced from Thumfart et al., 2011.

The first real mathematical model to bridge the gap between low-level statistical features and aesthetic emotions aroused by visual textures was proposed by Thumfart and colleagues [12]. Using the results of a psychological experiment, they modeled the relationship between computational texture features and their aesthetic properties. In contrast to previous approaches, this layered model provides insight into the hierarchical relationships involved in the aesthetic experience of texture properties. The structure of the hierarchical feed-forward model of aesthetic texture perception is shown in Figure 6. This model hypothesizes the presence of a hierarchical structure in the human aesthetic perception system. In fact, the existed findings indicate that aesthetic perception, appreciation and judgement require a distributed network of activation in regions associated with sensory, cognitive and motoric functions, and the related aesthetic experience taps into a comprehensive neural system, rather than into a single brain region [5]. The different regions of brain are connected with each other through complex neural circuits, which can be simplified into a hierarchical structure model. The research of Jacobs et al. demonstrated that the fusiform gyrus, the frontal operculum, occipitotemporal area, frontomedial cortex, and the amygdala are sensitive to beauty judgements [28]. In detail, the fusiform gyrus is sensitive to interactions between beauty level and type of judgement, which can be simplified into the affective layer as shown in Figure 6. The frontal operculum and occipitotemporal area appear responsive to the descriptive judgements. The frontomedial cortex and the amygdala appear to be selectively sensitive to beauty level during beauty judgements. Hence, these regions can be simplified into the judgement layer. The frontal and reward areas will make a final decision, which can be considered the emotional layer. Kirsch et al. proposed a schematic representation of the neural circuits implicated in aesthetic judgement, which were divided into visual areas, sensorimotor areas, and frontal and reward areas [4]. To some extent, the findings of Kirsch et al. are powerful support for the hierarchical feed-forward model of aesthetic texture perception.

To fully describe visual textures, 118 features are extracted, including three color features, 31 Gabor energy map features, 44 Fourier energy features, 10 neighborhood grey-tone difference matrix features, six Tamura features, and 88 grey-level cooccurrence features. Feature evaluation and selection decrease the model's complexity. In the original study, 27 pairs of intermediate aesthetic properties were selected, and six pairs of core aesthetic properties were assigned to three layers after psychological experiments. Two different approaches were used to develop the model. Supervised machine-learning methods automatically generate linear and nonlinear models when texture features and aesthetic properties are used as inputs and outputs, respectively. This model critically emphasizes the pathways in human perception that bridge the gap between low-level texture features and high-level aesthetic emotions triggered by those textures. Regression functions were used instead of classifiers to model human aesthetic perception of visual textures, and a continuous rating scale was employed to obtain reasonable data as output variables.

The major contribution of Thumfart and colleagues concerns the development of white-box models that allow for the identification of the interactions between aesthetic properties and features at different levels of complexity [12]. A structured regression model combines computational features and properties of low complexity to predict more complex aesthetic properties. However, the major limitation of the model is that only sixty-nine texture images were used in a semantic differential experiment to document the aesthetic properties.

**Figure 7:**
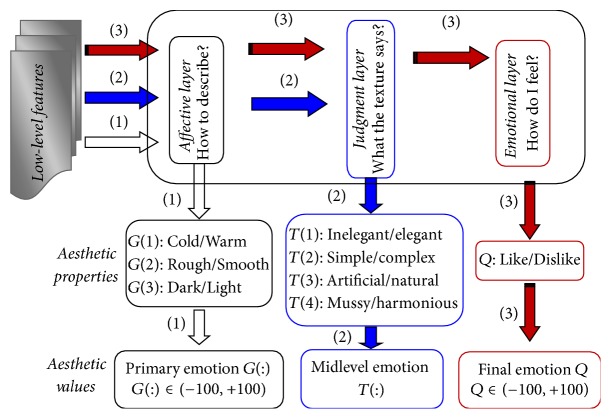
Hierarchical feed-forward dual-layered model, reproduced from Liu et al., 2015a and 2015b.

Similarly, Liu et al. built a hierarchical feed-forward three-layered model of aesthetic texture perception [13, 14]. The model proposed by Liu et al. is shown in Figure 7. Each layer has a set of interpretable aesthetic antonyms, with low-level features used as the original inputs. In addition to generating a predictive model for human aesthetic texture perception, Liu et al. also generated a structured model that allows for the interpretation of the relationships between low-level features and the aesthetic properties of visual textures. Liu et al. used multiple linear regression models to bridge the gap between low-level texture features and high-level aesthetic properties that are more interpretable and clearer in structure than black-box models, such as artificial neural networks.

Except for the six models mentioned above, some types of machine-learning models, such as deep learning, artificial neural network, and support vector machine, have been used for photograph aesthetical evaluation and prediction [57–62] Although the machine-learning models are black boxes, they are more convenient for model building because of their availability and generality with the development during the last decades. Nearly, all software programs for machine learning, such as MATLAB, Weka, and Enterprise Miner, provide neural network toolbox.

## 4. Discussion

In the present review, we discussed six individual models and the corresponding neural foundations for aesthetic perception. Although the internal structures of the models are very different, the aims of these models are similar. The relative advantages of these models of aesthetic perception are listed in Table 2.

As listed in Table 2, the first five models exhibit some structural parameters that are difficult to express using mathematical functions. To some extent, the neuropsychological model and the unifying model of visual aesthetic experience can be simplified into a hierarchical feed-forward model, such as those proposed by Thumfart and Liu [12–14]. However, the remaining three models remain too complex to translate into simple mathematical models. The mirror model of art focuses on the interface between the creation and appreciation of art, though the transformation of these complex processes into frameworks applicable for research purposes remains difficult. Similarly, the internal structures and the connections between each subsystem of the information-processing model and the quartet model of human emotion are complicated and cannot be expressed or quantified by mathematical functions or variables. However, neutrally based models of aesthetic perception may present a valuable tool for understanding the mechanisms underlying aesthetic appreciation. Nonetheless, the transformation of these complex, abstract models into simple mathematical functions remains a key focus of neuroaesthetics research.

What would qualify a model of aesthetic perception to be acceptable? This question can be difficultly answered before a mathematical model of aesthetic perception that truly human-like experiencing, perceiving, and appreciating has been built. Specifically, we argue that the acceptable models should do the following.

- The models support explanation and understanding of the theories of cognitive neuroscience, brain science, mathematics, and information science. The model is both descriptive and experimental, with qualitative observations and quantitative tests of hypotheses from the aspects of neuroscience and psychology.
- The models are compact enough with simple internal structures. To some extent, the simplicity of the model is preferable to the prediction performance when limited samples are used for model building and test. How do we make the simplest model the correct one? The biological architecture of visual cortex has provided a reference to simplify the aesthetics perception model into the hierarchical feed-forward structure like deep learning convolution networks. In other words, the combination of the strengths of recent neuroscience and brain science, especially the research results of neural underpinnings of visual aesthetic experience by using functional neuroimaging technology, is helpful to build a structured cognitive model of aesthetics perception.
- The models can be converted into mathematical ones with some conventional solving methods. The models should be white-box ones and can be formulized with some simple functions. The inputs and outputs are quantitative variables that can be gotten from psychology experiments and information-processing method. The general knowledge of artificial intelligence can be integrated into the aesthetics perception model to control the complexity and minimize structural risk and bad extrapolation behavior.

**Figure 8:**
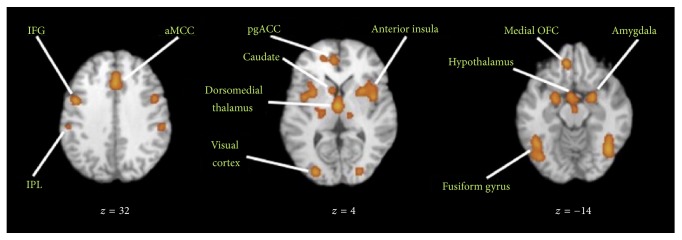
Functional connectivity of brain regions related to visual aesthetic perception. Reprinted from the article published by Steven Brown et al. [15]. IFG: inferior frontal gyrus; IPL: inferior parietal lobule; aMCC: anterior midcingulate cortex; pgACC: pregenual anterior cingulate cortex; OFC: orbitofrontal cortex.

**Figure 9:**
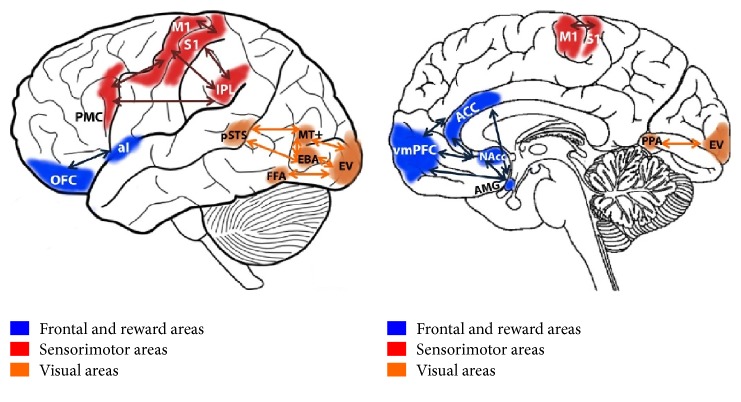
Schematic functional connectivity of the neural circuits implicated in aesthetic judgement tasks. Reprinted from the article published by Kirsch et al. [4]. OFC: orbitofrontal cortices; vmPFC: ventromedial prefrontal cortex; ACC: anterior cingulate; AMG: amygdala; aI: anterior insula; NAcc: nucleus accumbens; red parts: sensorimotor areas; M1: primary motor area; S1: primary somatosensory area; IPL: inferior parietal lobule; PMC: premotor cortex; orange parts: visual areas, part of the occipitotemporal cortex; EBA: extrastriate body area; MT: motion integration area; EV: early visual area; PPA: parahippocampal place area; pSTS: posterior superior temporal sulcus.

As mentioned in Section 3, the neural foundations of each model have been provided, although some of them are still under exploration stage without final validation. By summarizing the brain regions mentioned in each model, we can conclude that nearly all regions showing consistent activation to visual aesthetic experience as listed in Table 1 are mentioned. Using functional connectivity to posit the existence of neural networks for aesthetic perception of visual arts is a useful and common procedure. Regarding aesthetics neural circuits, Brown and collaborators proposed the existence of a core circuit for aesthetic processing in the brain, in which exteroceptive information passes through the orbitofrontal cortex (OFC) and interoceptive information passes through the anterior insula [15]. By reporting the results of voxel-based meta-analyses of 93 neuroimaging studies of positive-valence aesthetic appraisal of visual arts, Brown et al. found out that the most concordant areas of activation across visual aesthetic perception are the visual cortex, amygdala, hypothalamus, fusiform gyrus, anterior insula, medial OFC, and other correlated brain regions. The functional connectivity of these related brain regions is demonstrated in Figure 8. Additionally, Louise P. Kirsch et al. summarized the brain systems involved in aesthetic perception of fine arts and human body in the review, which is shown in Figure 9.

## 5. Conclusions

Aesthetic responses to beauty are not independent of the neural systems involved in sensory, perceptual, and cognitive processes. As suggested by Chatterjee and Vartanian, aesthetic experiences emerge from the interaction between neural systems involved in sensory-motor processes, emotion-valuation processes, and meaning-knowledge processes [63].

The models proposed by Chatterjee [6], Leder and colleagues [3], Tinio [9], Koelsch and colleagues [10], and Redies [11] imply that aesthetic appreciation and judgement are very complex and sophisticated cognitive processes that involve several different brain regions. Brain regions and neural connectivity associated with the perception of beauty have been explored using neuroimaging technology, providing strong support for neurobiological models of aesthetic appreciation. Although hierarchical feed-forward models [12–14] have sought to explore the relationship between computational texture features and aesthetic properties, the current model is unable to reveal the actual neural mechanisms underlying the aesthetic perception of visual texture when compared with those proposed by Chatterjee [6], Leder and Nadal [3], Tinio [9], Koelsch and colleagues [10], and Redies [11]. However, its simple internal structure is useful for controlling algorithm complexity and structural risk. Nonetheless, the development of a universal model of human aesthetic perception, appreciation, and judgement is underway in neuroaesthetics research.

All these efforts to understand the basis of aesthetic experience are not so that we can build up a mathematical model, but simply and hopefully to understand how this emerging property comes about. At best, the mathematics model could be a tool for exploration, not a constraint and lens through which to judge functional theories.

## Figures and Tables

**Table 1:** Regions showing consistent activation to visual aesthetic experience, corresponding to Brodmann areas [5].

Region	BA	Hem.
Parahippocampal gyrus	37	R
Culmen, anterior cerebellum		R
Fusiform gyrus	37	R
Middle frontal gyrus	46	R
Claustrum		R
Middle frontal gyrus	32	L
Inferior occipital gyrus	19	R
Parahippocampal gyrus	36	L
Parahippocampal gyrus	37	L
Inferior frontal gyrus	9	L
Middle frontal gyrus	6	R
Insula	13	L
Inferior frontal gyrus	9	R
Precuneus	7	R
Parahippocampal gyrus	27	R
Amygdala		R
Inferior occipital gyrus	19	L
Middle occipital gyrus	18	L
Lingual gyrus	18	R
Lingual gyrus	18	L
Inferior occipital gyrus	18	R
Anterior cingulate cortex	32	L
Anterior cingulate cortex		R
Parahippocampal gyrus	27	L
Precentral gyrus	4	L
Amygdala		L
Amygdala		L

**Table 2:** Comparative analysis of the six reviewed models.

Model name	Objective of the model	Input stimulus	Are there some structural parameters that cannot be quantified for the development of mathematical models?
Neuropsychological model [6]	The neuropsychological model is derived from visual cognitive neuroscience and aims to propose a general framework for the neural underpinnings of visual aesthetics and to explain how visual stimuli are mapped to emotions.	Visual information	Nearly all the visual computation stages involved in this model can be mathematically simulated. There are some models and algorithms for visual feature extraction. This model is easily acceptable for model building.

Information-processing model [7, 8]	By positing several distinctive processing stages among cognitive and emotional processes, information-processing models have been proposed for empirical research on the perception of art and aesthetic experiences.	Modern and contemporary visual art, everyday objects, design objects, dance and body perception, music, and food	Some structural parameters, such as the time course of early processes, the relevance of prior experience, the complexity and relevance of emotional processes, and life-relevant experiences, are difficult to quantify.

Mirror model of art [9]	The mirror model of art focuses on the interface between the creation and appreciation of art. The model is based on the assumption that the fundamental nature of art-making and art-viewing is related.	Visual arts	The knowledge and background experiences of the artists and perceivers when they are creating and viewing art are difficult to describe with computational variables.

Quartet model of human emotion [10]	The quartet model of human emotion presents a neurobiological theory of emotions and proposes four core emotional systems. Aesthetic perception is also covered in the quartet model.	Everything related to emotion, including language	The quartet model of human emotion focuses on the interactions and connections among the four core systems. How to model the interactions among these four systems using mathematical functions is a great challenge.

Unifying model of visual aesthetic experience [11]	The model aims to explain factors involved in aesthetic experiences in response to visual artworks.	Visual stimuli	The forward flow of information in different stages and the encoding of information in the nervous system can be expressed by mathematical functions. However, the beauty-responsive mechanism and the personal cultural filter are difficult to describe using mathematical models.

Hierarchical “feed-forward” model [12–14]	The hierarchical feed-forward model was developed using multiple linear regression to investigate the relationship between human aesthetic texture perception and computational low-level texture features. These white-box models can be interpreted in terms of both structure and interactions between aesthetic properties and texture features according to feature weights.	Visual stimuli	The models can be expressed with mathematical functions. However, the models do not accurately reflect the real functions of the neural systems involved in the aesthetic perception of visual art.
